# A new marine cyclopoid copepod of the genus *Neocyclops* (Cyclopidae, Halicyclopinae) from Korea

**DOI:** 10.3897/zookeys.520.6006

**Published:** 2015-09-16

**Authors:** Jimin Lee, Cheon Young Chang

**Affiliations:** 1 Biological Oceanography & Marine Biology Division, Korea Institute of Ocean Science & Technology, Ansan, Korea; 2 Department of Biological Science, College of Natural Sciences, Daegu University, Gyeongsan, Korea

**Keywords:** Beach, description, interstitial, meiofauna, northwest Pacific, taxonomy

## Abstract

A new cyclopoid species of the genus *Neocyclops* Gurney, 1927 is described. Type specimens were collected from a beach on south-western coast of the Korean Peninsula by rinsing intertidal coarse sandy sediments. *Neocyclops hoonsooi* **sp. n.** is most characteristic in showing the conspicuous chitinized transverse ridges originating from the medial margins of the coxae of all swimming legs. The new species is most similar to *Neocyclops vicinus*, described from the Brazilian coast, and *Neocyclops petkovskii*, from Australia. All three species share a large body size (more than 750 µm long), the presence of an exopodal seta on the antenna, two setae on the mandibular palp, the same seta/spine armature on the third endopodal segment of leg 3 (3 setae + 3 spines), and the fairly long inner distal spine on the third endopodal segment of the female leg 4. However, *Neocyclops hoonsooi* **sp. n.** differs from both species by the much shorter caudal rami (less than 1.7 times as long as wide) and the shorter dorsal caudal seta VII. Furthermore, *Neocyclops hoonsooi* is clearly distinguished from *Neocyclops vicinus* by the 10-segmented antennule (vs 12 segments in *Neocyclops vicinus*), and from *Neocyclops petkovskii* by the elongate inner distal spine on leg 5 exopod and the 3-segmented leg 5 in male (vs 4-segmented in *Neocyclops petkovskii*). A tabular comparison of characters separating *Neocyclops hoonsooi* from its closest allies and a key to *Neocyclops* species from the Indo-Pacific Ocean are provided. This is the first record of the genus *Neocyclops* from the northern Pacific.

## Introduction

Members of the genus *Neocyclops* Gurney, 1927 (Cyclopidae, Halicyclopinae) typically inhabit marine epibenthic or interstitial environments. The genus is widely distributed in coastal, surface and subterranean (anchialine) habitats of the Northeast and Tropical Atlantic, the Mediterranean, the Black and Red Seas and the Indo-Pacific (West Australia, Papua New Guinea), with an endemism index of about 95% (Pesce 2015). Karanovic (2008) recently revised the genus and added 11 new species, including eight interstitial species from Australia. However, notwithstanding its wide distribution and potentially high diversity, the taxonomy of the genus is in a state of disarray. The primary reasons for this state of affairs are the paucity of useful diagnostic characters to differentiate most species (due to the very conservative morphology within the genus), the significant variability displayed by some of them and the inadequate descriptions of several previously described species (Karanovic 2008). The genus currently accommodates 24 nominal species but it is known that many as yet unnamed species await description (Karanovic 2008, Walter and Boxshall 2015, Pesce 2015).

Although cyclopoid copepods constitute an important component of the marine epibenthic and interstitial fauna, our knowledge of their taxonomy and diversity is relatively very poor in comparison with freshwater cyclopoids, especially in the northwest Pacific region (Chang 2011, Karanovic 2014). Chang (2011) recorded a new species of the genus *Cyclopinoides* Lindberg, 1953 (Smirnovipinidae) from the Korea Strait (Tsushima Island, Japan and Busan, Korea), and recently Karanovic (2014) described a new species of *Euryte* Philippi, 1843 (Cyclopidae) from the East Sea (Sea of Japan). During field surveys of the marine interstitial cyclopoids from Korea, a new species of the genus *Neocyclops* was found in a beach on the south-western coast of the Korean Peninsula, representing the first record of the genus from the North Pacific. In this paper we provide a detailed illustrated description of both sexes, including a tabular comparison of the salient characters distinguishing the new species from its closest congeners.

## Materials and methods

Collections were made at Holtong beach, located along the south-western coast of the Korean Peninsula, in shallow littoral (about 0.5–1 m deep) by scooping the surface layer of a coarse sand bottom with a long-handled dipper. Sediment samples were gathered into a bucket, subjected to freshwater shock and filtered through a conical plankton net or plankton hand-nets (mesh size 64 µm). Samples were immediately fixed in the field by adding a few drops of 35% formaldehyde. Copepods were sorted in the laboratory, using a micropipette under a zoom-stereomicroscope (Zeiss SV-11, Germany), and transferred to 80% ethanol or to 4% buffered formaldehyde for long-term preservation.

Methods for dissection, double-coverglass preparation using H-S slides (see Shirayama et al. 1993), drawing and measurements followed those outlined in Chang (2013, 2014).

Type specimens are deposited in The Natural History Museum, London (NHMUK) and the specimen room of the Department of Biological Science, Daegu University (DB), Korea.

General terminology for the description of the new species follows Huys and Boxshall (1991). Abbreviations used in the text, table and figure legends follow the conventional ones frequently used in the taxonomy of copepods: enp-1 to enp-3 or exp-1 to exp-3, the first to third endopodal or exopodal segments of each leg. Sewell’s (1949) system is adopted for seta/spine armature of legs 1–4, where setae are denoted by Arabic numerals, and spines by Roman numerals (Tran and Chang 2013, for details cf. Huys and Boxshall 1991, fig. 1.5.7).

## Systematic accounts

### Family Cyclopidae Rafinesque, 1815 Genus *Neocyclops* Gurney, 1927

#### Neocyclops hoonsooi sp. n.

Taxon classificationAnimaliaCyclopoidaCyclopidae

http://zoobank.org/451980E9-A08A-4365-8240-7A185C4877C6

Figs 1 , 2 , 3 , 4

##### Type locality.

Holtong beach (35°03.68'N, 126°19.87'E), South Korea, Jeollanam-do Province, Muan-gun County, Hyeongyeon-myeon, Oryu-ri; western coast of the Dadohae Oceanic National Park, South Korea.

##### Material examined.

Holotype ♀ (DB20046), allotype ♂ (DB20047), both dissected on slides. Paratypes: 1♀ (NHMUK reg. no. 2015. 3056), 1♂ (NHMUK reg. no. 2015. 3057), both undissected, ethanol-preserved; 2♀♀ (DB20048, 20049), 1♂ (DB20050), dissected on slides; 2♀♀ (DB20051), 2♂♂ (DB20052), in ethanol. All specimens were collected from the type locality by J. Lee on 21 August 2008.

##### Diagnosis.

Female habitus large, about 830 µm long. Genital double-somite with lateral expansions in anterior quarter. Caudal rami about 1.7 times as long as wide, with 7 caudal setae, including vestigial ventrolateral seta I; inner caudal seta VI well developed, about 1.7 times longer than outer caudal seta III; dorsal seta VII slightly shorter than caudal ramus. Antennule 10-segmented. Antenna with exopodal seta. Exp-3 of legs 1–4 with setal formula 5,5,5,5 and spine formula 3,4,4,3; enp-3 of leg 3 bearing 3 spines and 3 setae; inner distal spine on enp-3 of leg 4 distinctly longer than enp-3 and outer distal spine. Leg 5 exopod subpyriform, about twice as long as wide; inner distal spine 1.2 times longer than outer spine, about 1.4 times as long as lateral spine, about 0.9 times as long as exopod. Male caudal rami 1.36 times longer than wide, with 7 caudal setae. Male leg 5 3-segmented, comprising coxa, basis and exopod.

##### Description.

**Figure 1.**
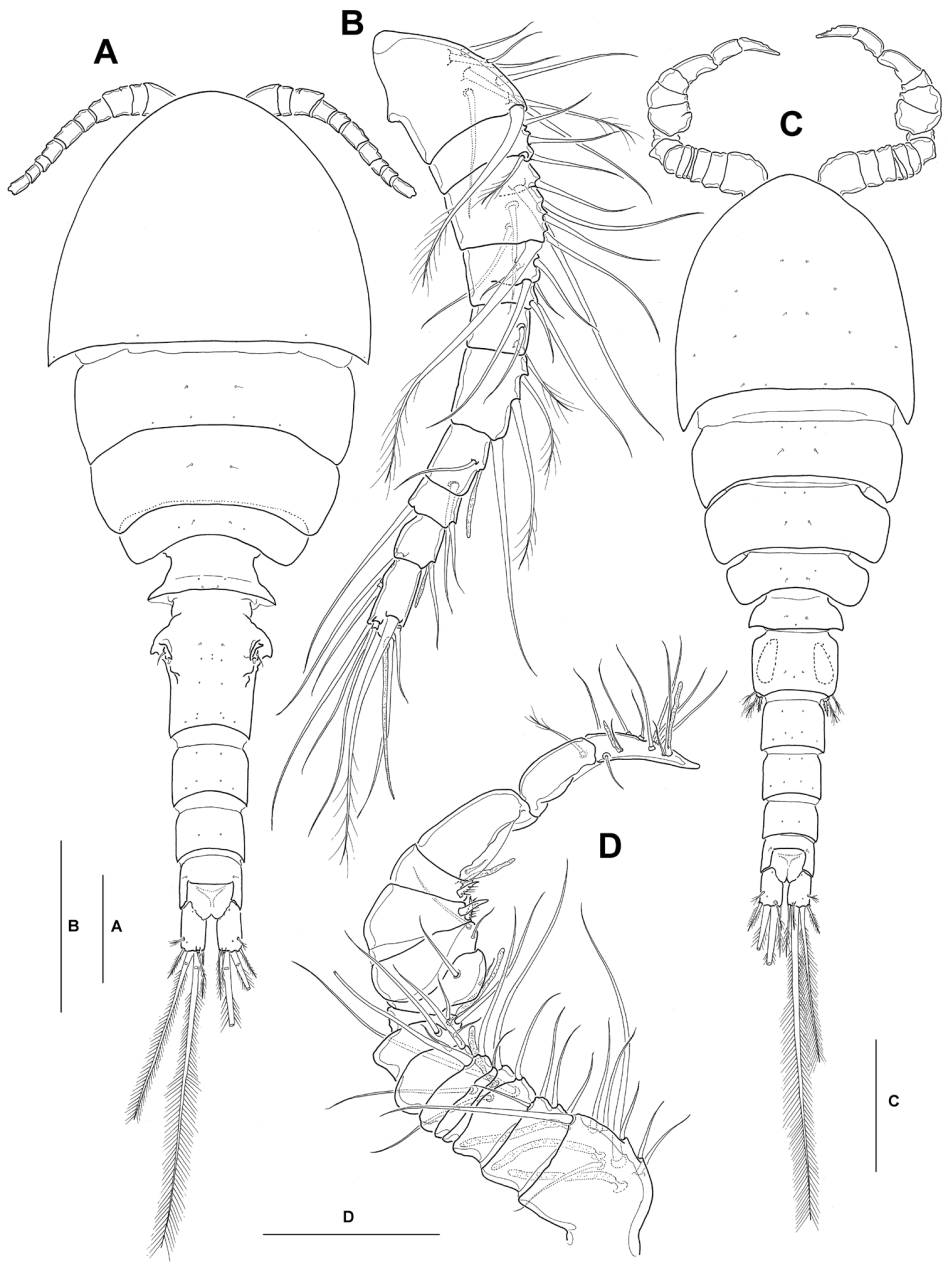
*Neocyclops hoonsooi* sp. n. **A–B** female: **A** habitus, dorsal **B** right antennule, dorsal. **C–D** male: **C** habitus, dorsal **D** left antennule, dorsal. Scale bars: 100 µm (**A**, **C**), 50 µm (**B**, **D**).

Female (Holotype). Body (Fig. 1A) large and robust, 830 µm long, (mean 826 µm, standard deviation 12, *n* = 6), excluding rostrum and caudal setae. Body width 303 µm, greatest width at posterior margin of cephalothorax; body length/width ratio about 2.7. Color of preserved specimens a milky white tinge.

Prosome oval, about 1.2 times longer than urosome, a little protruding anteriorly. Rostrum reflexed downwards, not discernible in dorsal view, with blunt apex in ventral aspect. Nauplius eye not discernible. Cephalothorax not strongly protruding anteriorly, slightly longer than 4 free thoracic somites combined; first pedigerous somite completely incorporated into cephalosome. Prosomites not showing pronounced lateral expansions, with narrow and nearly smooth hyaline fringe along posterior; ornamented with one pair of minute sensilla medially on dorsal surface of second to fourth pedigerous somites, and a few integumental pores near posterior margin of each prosomite.

**Figure 2.**
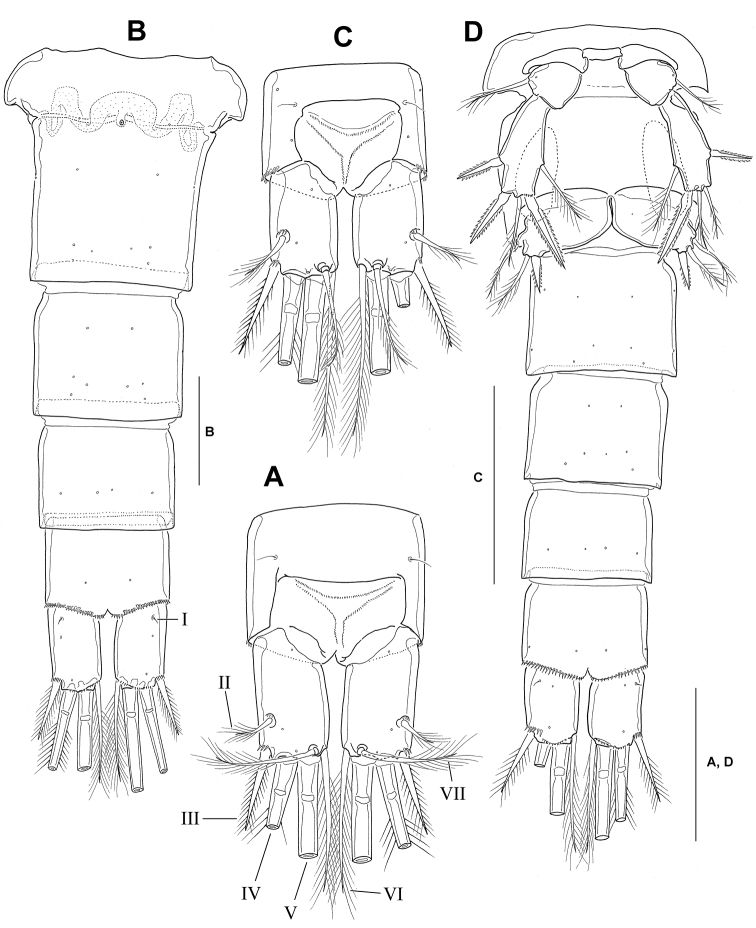
*Neocyclops hoonsooi* sp. n. **A–B** female: **A** urosome, ventral **B** anal somite and caudal rami, dorsal. **C–D** male: **C** anal somite and caudal rami, dorsal **D** urosome and leg 5, ventral. Scale bars: 50 µm.

Urosomites (Figs 1A, 2B) length ratios, beginning with fifth pedigerous somite, 36: 100: 43: 39: 43; with hyaline membrane along posterior margins both dorsally and ventrally; spinule rows lacking, except for anal somite with about 20 minute spinules along ventral posterior margin; arrangement of cuticular pores as shown in Figs 1A, 2A and 2B. Fifth pedigerous somite slightly narrower than genital double-somite, ornamented with paired middorsal sensilla; posterolateral corner pronounced. Genital double-somite slightly longer than broad, with paired backwardly directed spinous processes in anterior quarter; leg 6 represented by one seta and one small cuticular projection surrounded by cuticular wrinkles dorsolaterally. Copulatory pore small, located midventrally in about proximal quarter of genital double-somite; seminal receptacle fully fused medially; both lateral sides transversely undulating, as shown in Fig. 2B. Anal somite much shorter than wide, about 3/4 times longer than caudal rami; 1 pair of dorsal sensilla just anterior to lateral corners of anal operculum. Anal operculum (Fig. 2A) situated at halfway the anal somite length, not strongly convex with smooth posterior margin.

Caudal rami (Fig. 2A, B) nearly parallel, with 7 setae; ramus 1.68 times (ranging from 1.64 to 1.72, standard deviation 0.04, *n* = 6) longer than wide, in ventral view, slightly shorter than anal somite; dorsal and medial surfaces of rami smooth, without hairs along inner (medial) margin; outer margin nearly smooth, not interrupted by indentations or spinules. Anterolateral seta I vestigial, represented by minute setule, situated in anterior part of ventral surface (Fig. 2B). Lateral seta II located slightly dorsally, issuing from about distal quarter of outer margin of ramus. Outer seta III short, spiniform and bipinnate, about 0.7 times as long as ramus, a little less than 2/3 length of inner seta VI, surrounded by 3–5 minute spinules at base. Terminal setae IV–V with fracture planes, bipinnate. Inner seta VI well developed, plumose, about 1.2 times as long as caudal rami, about 1.7 times longer than outer seta III. Dorsal seta VII slender, plumose, about 2/3 times as long as inner seta VI, and slightly shorter (0.9 times) than caudal ramus.

Antennule (Fig. 1B) short, reaching to about middle of cephalothorax; 10-segmented; segments 3 and 5 with incomplete ventral and dorsal sutures, respectively, indicating original subdivision. Setal formula: 1-[8], 2-[4], 3-[2+6], 4-[4+2], 5-[2], 6-[3], 7-[2+1 aesthetasc], 8-[2], 9-[2+1 aesthetasc], 10-[7+1 aesthetasc].

**Figure 3.**
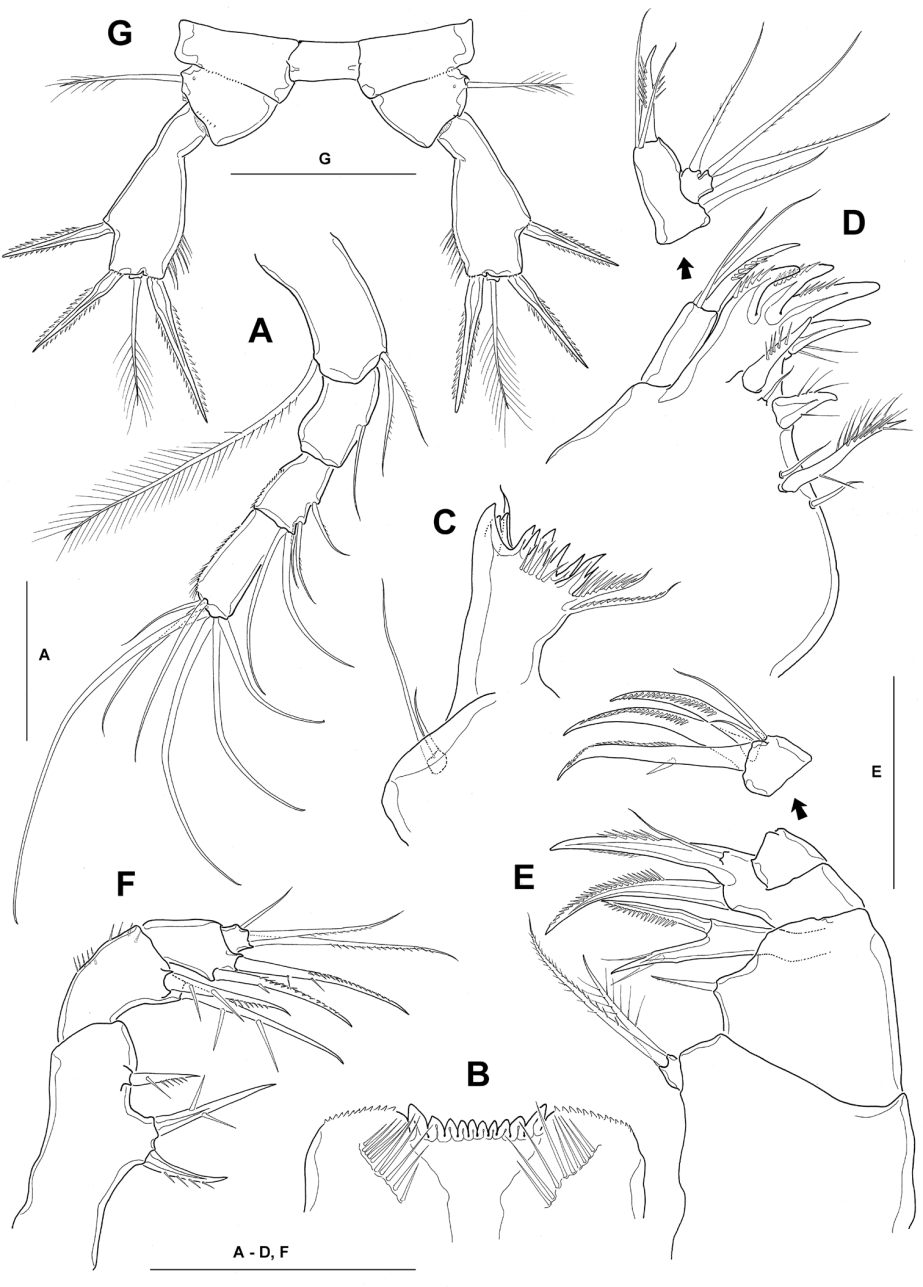
*Neocyclops hoonsooi* sp. n., female. **A** antenna **B** labrum **C** mandible **D** maxillule **E** maxilla **F** maxilliped **G** leg 5. All scale bars: 50 µm.

Antenna (Fig. 3A) slender, distinctly 4-segmented, comprising coxobasis and 3-segmented endopod. Coxobasis about 2.1 times as long as wide, with 1 long outer seta distally (unipinnate proximally and plumose distally), representing exopod, and 2 unipinnate setae at inner distal corner. First endopodal segment about 1.7 times as long as wide, with 1 naked seta at halfway the inner margin. Second endopodal segment small, about 1.5 times as long as wide, with minute spinules along outer margin; armed with 1 short medial, 2 short subapical and 2 long apical setae along inner margin. Third endopodal segment elongate, about 2.5 times as long as wide, ornamented with 1 spinular row along outer margin, bearing 7 apical setae including 4 geniculate and 3 slender setae.

Labrum (Fig. 3B) trapezoidal, armed with 10 strong teeth on broad, slightly concave cutting edge; serrated along distolateral margins; posterior surface with 2 oblique rows of 9–10 long, slender spinules.

Mandible (Fig. 3C), palp reduced to small protuberance, bearing 2 slender, naked setae apically; longest seta not reaching to gnathobasal teeth, about 3 times as long as shorter one. Coxal gnathobase well-developed; cutting edge armed with innermost complex of 3 stout teeth and 1 spinous element, middle group of 6 teeth and 5 sharp spinules, and outer group of 1 unipinnate spine and 1 outer subapical unipinnate seta.

Maxillule (Fig. 3D) comprising well developed praecoxa and 2-segmented palp. Praecoxa armed with 4 strong dentate spines inner distally, composed of 3 unipinnate spines basally fused together, and 1 separated posteriormost spine; 6 elements situated along inner face, consisting of 2 strong spinous setae, 1 longest pinnate seta and 3 small, naked setae. Maxillular palp completely divided, about 1.8 times as long as its greatest width, bearing 1 strong bipinnate spine and 2 slender, naked setae distally; endopod small, bearing 1 lateral and 2 apical setae, flanked by 1 proximal seta representing exopod.

Maxilla (Fig. 3E) 4-segmented (praecoxa and coxa fused on posterior surface). Praecoxa with distal endite bearing 1 pinnate and 1 plumose setae apically; proximal endite reduced and unarmed. Coxa, proximal endite represented by 1 short, minutely pinnate seta; distal endite highly mobile, armed with 1 strong, basally fused, spinous element, bearing 2 setules distally, and 1 strong, unipectinate, spinous element. Basis forming a bipinnate claw, with one 1 slender, naked seta at base; 1 strong, unipectinate, spinous element curved, slightly longer than claw. Endopod slightly tapering distally, armed with 3 long, curved, unipinnate, spinous elements and 2 naked setae.

Maxilliped (Fig. 3F) slender, 4-segmented, comprising syncoxa, basis and 2-segmented endopod. Syncoxa, about 2.2 times as long as broad, unornamented; medial margin with 2 endites, bearing 2 and 1 strong, spinous setae, respectively. Basis about 1.7 times as long as broad, with group of spinules halfway outer margin and near outer distal corner; bearing 2 spinous setae inner distally, each with 2 long secondary spinules on posterior margin. First endopodal segment unornamented, with 2 pinnate inner setae; second endopodal segment small and subquadrate, with 1 short, subapical and 2 long, apical setae.

**Figure 4.**
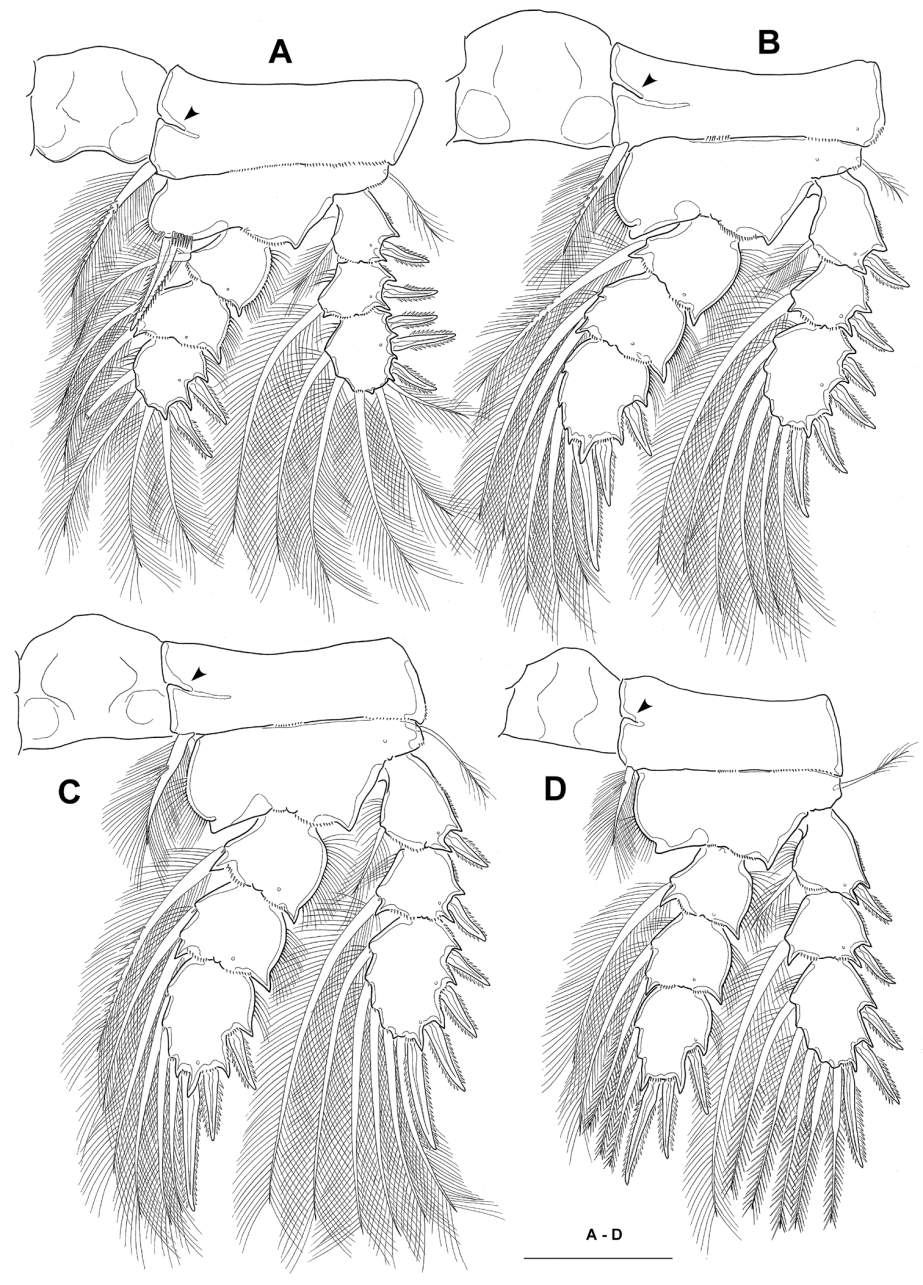
*Neocyclops hoonsooi* sp. n., female. **A** leg 1 **B** leg 2 **C** leg 3 **D** leg 4. All scale bars: 50 µm.

Legs 1–4 (Fig. 4A–D) biramous, both rami 3-segmented. Intercoxal sclerites of legs 1–4 with smooth distal margin, each with 2 lateral lobes, those of leg 1 most pronounced, unarmed with smooth distal margins, not ornamented with any transverse setule or spinule row on both frontal and caudal surfaces. Praecoxal sclerites not expressed. Coxae unornamented, except for spinule row on posterior margin; with transverse internal chitinous ridges originating from medial margins; inner distal plumose seta well developed, but that of leg 4 conspicuously shorter. Exp-3 of legs 1–4 with setal formula 5,5,5,5 and spine formula 3,4,4,3; each leg bearing 2 inner setae on enp-2, and 1 inner seta on enp-1 and exp-1. Leg 1 (Fig. 4A), intercoxal sclerite not broad, its free margin concave; inner distal seta of basis remarkably stout, bipinnate, its tip nearly reaching to distal margin of enp-2. Leg 4 (Fig. 4D), free margin of intercoxal sclerite smooth and nearly straight; enp-3 1.24 times longer than wide; inner distal spine 1.14 times longer than enp-3, 1.36 times longer than outer distal spine; inner setae on exp-3 and enp-3 with swollen proximal half and slender distal half. Seta/spine armature of legs 1–4 as follows:

**Table:** Seta/spine armature of legs 1–4.

	coxa	basis	exopod	endopod
Leg 1	0-1	1-1	I-1, I-1, III,2,3	0-1, 0-2, I,I+1,3
Leg 2	0-1	1-0	I-1, I-1, III,I+1,4	0-1, 0-2, II,I,3
Leg 3	0-1	1-0	I-1, I-1, III,I+1,3	0-1, 0-2, II,I,3
Leg 4	0-1	1-0	I-1, I-1, II,I+1,4	0-1, 0-2, I,II,2

Leg 5 (Fig. 3G) 3-segmented; intercoxal sclerite quadrangular, about twice as long as wide, with nearly straight posterior margin, lacking spinule ornamentation. Coxa clearly defined from fifth pedigerous somite; about twice as wide as greatest length (measured along inner margin); armed with 1 row of minute spinules along distal margin. Basis subtriangular, about 1.5 times wider than long, with 1 plumose seta laterally; 1 cuticular pore present near base of lateral seta. Exopod subpyriform, about twice as long as wide; inner margin tapering abruptly in proximal 1/5, and gradually broadening distally, then slightly narrowing with inner setule row in distal quarter; bearing 2 apical, bipectinate spines flanking 1 long plumose seta apically and 1 subapical spine in distal third of outer margin; inner distal spine 1.2 times longer than outer spine, about 1.4 times as long as lateral spine, about 0.86 times as long as exopod.

Male (allotype): Body (Fig. 1C) 564 µm long (mean 572 µm, standard deviation 10, *n* = 5). Caudal rami (Fig. 2C, D) 1.36 times longer than wide (conspicuously shorter than in female), with similar setal armature as in female.

Antennule (Fig. 1D) 16-segmented; strongly modified, digeniculate, with major geniculation between segments 14 and 15 and secondary geniculation between segments 8 and 9; segments 14–15 cuticular folds along anterior margin; segment 16 claw-like and curved, with 2 short aesthetascs along posterior margin. Aesthetasc formula: 3,0,0,1,0,0,0,0,1,0,0,0,1,0,0,2. Two elements on anterior margins of segments 12–13 short and spiniform; 1 ventral seta on segment 15 plumose; other setae naked, slender.

Leg 5 (Fig. 2D) 3-segmented, with small intercoxal sclerite; coxa unarmed; basis with slightly swollen inner margin, bearing 1 outer plumose seta, with 1 cuticular pore near base of lateral seta. Exopod about 2.3 times as long as wide; seta/spine armature similar to that in female, except for additional seta on inner margin; allotype showing aberrant asymmetrical spine armature on left side, with outer apical spine being replaced by 1 short plumose seta (see Fig. 2D). Leg 6 reduced to operculum with 1 short inner bipinnate spine and 2 plumose setae distally; outer seta slightly longer than inner seta.

##### Etymology.

The proposed specific name is dedicated to the late Professor Hoon Soo Kim in honor of his contribution to the development of invertebrate taxonomy in Korea.

##### Ecology.

This species was found at Holtong beach along the western coast of the Dadohae Oceanic National Park, which is located along south-western coast of the Korean Peninsula. The beach is exposed and fringed with rocks on both sides. The intertidal, coarse to medium sandy sediments contained a little mud. Salinity: 27–32 ‰. The new species co-occurred with other interstitial ones: *Cerconeotes japonicus* (Itô, 1968), *Cyclopina* spp. (Copepoda), *Xenotrichula* sp. (Gastrotricha), and *Echinoderes* sp. (Kinorhyncha).

##### Remarks.

The genus *Neocyclops* currently accommodates 24 nominal species (Karanovic 2008, Pesce 2015, Walter and Boxshall 2015). Petkovski (1986) proposed a division into two subgenera according to the number of segments in the male leg 5, *i.e.* 4 segments in the subgenus Protoneocyclops Petkovski, 1986 and 3 segments in the subgenus Neocyclops Gurney, 1927. This classification has not been universally accepted yet (for details, see Karanovic 2008). Based on the presence of a 3-segmented leg 5 in the male, *Neocyclops hoonsooi* sp. n. might be allocated to the nominotypical subgenus *Neocyclops* which includes 11 species at present. Nine *Neocyclops* species which show the 4-segmented condition in the male are currently assigned to the subgenus *Protoneocyclops*, while four other species are known from females only and can as yet not be attributed to either subgenus: *Neocyclops parvus* (Sewell, 1949), *Neocyclops magnus* (Sewell, 1949), *Neocyclops improvisus* Pleşa, 1973 and *Neocyclops sharkbayensis* Karanovic, 2008.

*Neocyclops* species typically possess 12-segmented antennules in the female but a few members are known to show fewer segments: 11-segmented in *Neocyclops improvisus* and *Neocyclops geltrudeae* Pesce & Galassi, 1993, 10-segmented in *Neocyclops petkovskii* De Laurentiis, Pesce & Halse, 1997, and only 8-segmented in *Neocyclops salinarum* (Gurney, 1927). *Neocyclops hoonsooi* sp. n. shares the 10-segmented condition with *Neocyclops petkovskii*, showing traces of subdivision in compound segments 3 and 4. *Neocyclops hoonsooi* sp. n. also shares with *Neocyclops petkovskii* an exopodal seta on the antenna, which is absent in *Neocyclops parvus*, *Neocyclops medius* Herbst, 1955, *Neocyclops affinis* (Pleşa, 1961), *Neocyclops improvisus*, *Neocyclops australiensis* Karanovic, 2008, and *Neocyclops dussarti* Karanovic, 2008 [= *nomen novum* for *Neocyclops affinis* Dussart, 1974, a junior homonym of *Neocyclops affinis* (Pleşa, 1961), for details, see Karanovic 2008: 262].

The new species is most characteristic in having large scar-like integumental ridges originating from the medial margins of the coxae in all swimming legs. The transverse chitinized reinforcements are very conspicuous, and consistently occurred in all specimens examined. Similar structures have been illustrated for three species that were recently described from Australia by Karanovic (2008): figure 54A and D for leg 1 and leg 4 of *Neocyclops australiensis*, figures 58D and 59B for leg 1 and leg 4 of *Neocyclops sharkbayensis*, and figure 61C for leg 3 of *Neocyclops trajani* Karanovic, 2008. However, all of them are less pronounced, and illustrated as small open-circles or ovals in close connection to the medial margin of the coxae, which showed quite different patterns from those of the new species.

The new species is also unusual in bearing a small setule on the anteroventral surface of the caudal ramus in both sexes. This setule is here identified as the anterolateral accessory seta I. As far as we can ascertain, it was recorded only once before in the genus *Neocyclops*, *i.e.* in the description of the female caudal ramus of *Neocyclops pilbarensis* Karanovic, 2008, where it was interpreted as a “sensillum at anterior part ventrally”. While the caudal seta I is rarely expressed and usually lacking in members of the Cyclopoida, it can sometimes be quite conspicuous in some marine, and especially ancestral, genera, such as *Heterocyclopina* Pleşa, 1968. Karanovic (2008, fig. 49A, B) interpreted a similar structure as the “lateral sensillum” in his description of *Abrsia misophrioides* but did not consider the possibility of it being one of the caudal setae. Based on positional homology we believe that the “sensillum” observed in *Neocyclops pilbarensis* and *Abrsia misophrioides* represents the vestigial caudal seta I and is homologous with the minute seta described in *Neocyclops hoonsooi* sp. n.

Another unusual characteristic of *Neocyclops hoonsooi* sp. n. is the very short caudal ramus, being slightly less than 1.7 times as long as wide. Caudal rami of *Neocyclops* species are generally more than twice as long as wide, being about 2.0–2.5 times in *Neocyclops affinis*, *Neocyclops parvus*, *Neocyclops australiensis* and *Neocyclops ferrarii* Rocha, 1995, 2.7–3.0 times in *Neocyclops magnus* and *Neocyclops vicinus* (Herbst, 1955), and even reaching to 3.5–4.0 times in *Neocyclops remanei* (Herbst, 1952). However, in a few species the caudal ramus is much shorter, and less than twice as long as wide, being about 1.8–2.0 times in *Neocyclops medius* and *Neocyclops dussarti*, and slightly less than 1.7 times in *Neocyclops hoonsooi* sp. n. Two genuinely interstitial species from beaches in southern Australia, have extremely short caudal rami (1.5 times in *Neocyclops tropicus* Karanovic, 2008, and 1.3 times in *Neocyclops trajani*), however, these species differ clearly from *Neocyclops hoonsooi* sp. n. by the much smaller body size (546–565 µm long), the 12-segmented antennule, the presence of 3 setae on the mandibular palp, and the very long dorsal caudal seta (1.5–2.4 times longer than caudal rami). The caudal seta VII in *Neocyclops hoonsooi* sp. n. is slightly shorter or nearly as long as the caudal ramus. This condition is shared with *Neocyclops ferrarii*, *Neocyclops improvisus*, *Neocyclops magnus*, *Neocyclops mediterraneus* (Kiefer, 1960), *Neocyclops remanei* and *Neocyclops vicinus*, while most other species have a much longer dorsal seta (more than twice longer than the caudal ramus): *Neocyclops geltrudeae*, *Neocyclops pilbarensis*, *Neocyclops sharkbayensis* and *Neocyclops tropicus*. *Neocyclops papuensis* Fiers, 1986 clearly differs from all its congeners, including the present new species, by bearing an extremely short dorsal seta (0.4 times as long as the caudal ramus). Seta VI (innermost caudal seta) of *Neocyclops hoonsooi* sp. n. is much longer than seta III (outermost caudal seta), and thus differs from those species that display the reverse condition (seta III longer than seta VI) such as *Neocyclops affinis*, *Neocyclops vicinus*, *Neocyclops improvisus*, *Neocyclops monchenkoi* Karanovic, 2008 and *Neocyclops australiensis*.

*Neocyclops hoonsooi* sp. n. displays the typical seta/spine armature pattern on legs 1–4 found in the majority of species in the genus *Neocyclops*. The setal formula of the third exopodal segments of the new species is 5,5,5,5, which differs from the 5,5,5,4 pattern in *Neocyclops herbsti* Petkovski, 1986 and the 4,5,5,5 condition in *Neocyclops wellsi* Petkovski, 1986. The spine formula of the third exopodal segments of *Neocyclops hoonsooi* sp. n. is 3,4,4,3, and differs only from the 2,4,4,3 pattern of *Neocyclops sharkbayensis*. The setal formula on the distal endopodal segments of the new species is 4,3,3,2, and differs from the 4,3,4,2 pattern displayed by *Neocyclops affinis*, *Neocyclops dussarti* and *Neocyclops improvisus* (Pleşa 1961, 1973, Dussart 1974). The spine formula of the third endopodal segments of *Neocyclops hoonsooi* sp. n. is 2,3,3,3, and differs from the 2,3,2,3 condition observed in *Neocyclops monchenkoi*. *Neocyclops* species, including the new species, typically bear two setae along the inner margin of the second endopodal segment of all swimming legs; the only exception to this rule is *Neocyclops sharkbayensis* which displays a single seta only on legs 1–2.

Taking into consideration the characters mentioned above, *Neocyclops hoonsooi* sp. n. appears to be most similar to *Neocyclops vicinus*, described from the Brazilian coast, and *Neocyclops petkovskii*, from Australia. All three species share a large body size (more than 750 µm long), the presence of an exopodal seta on the antenna, two setae on the mandibular palp, the same seta/spine armature on the third endopodal segment of leg 3 (3 setae + 3 spines), and the fairly long inner distal spine on the third endopodal segment of the female leg 4. However, *Neocyclops hoonsooi* sp. n. clearly differs from *Neocyclops vicinus* by the following characters: (1) 10-segmented antennule (vs 12 segments in *Neocyclops vicinus*); (2) shorter caudal rami (less than 1.7 times as long as wide, while about three times longer in *Neocyclops vicinus*); and (3) much shorter dorsal caudal seta VII (about 2/3 times shorter than inner caudal seta VI, while 1.4 times longer in *Neocyclops vicinus*), and much longer inner caudal seta VI (more than 1.5 times longer than outer caudal seta III, while slightly shorter than outer one in *Neocyclops vicinus*). Furthermore, *Neocyclops hoonsooi* sp. n. also clearly differs from *Neocyclops petkovskii* by the much shorter caudal rami (vs 2.4 times as long as wide in *Neocyclops petkovskii*), the shorter inner distal spine on the female leg 5 (vs slightly shorter than the outer distal and lateral spines, and about half the length of the exopod in *Neocyclops petkovskii*), and the 3-segmented leg 5 in male (vs 4-segmented in *Neocyclops petkovskii*). Table 1 shows the character comparison between the new species from South Korea and its closest allies.

**Table 1.** Character comparison among the allied species of *Neocyclops hoonsooi* sp. n.

	*affinis*	*australiensis*	*improvisus*	*monchenkoi*	*petkovskii*	*vicinus*	*hoonsooi* sp. n.
♀, body length (µm)	390–439	731	396–488	720–1,110	765	750	830
♀, antennule, no. of segments	12	12	11	12	10	12	10
Antenna, exp seta	absent	absent	absent	present	present	present	present
Mandible, no. of setae on palp	1	3	1	2	2	2	2
Caudal rami, L/W ratio,♀	2.2–2.6	2.5	1.8–2.0	2.7–3.5	2.4	~ 3	1.6–1.7
Length ratio, caudal setae VI/III	~ 1	0.7	0.9	~ 0.9	~ 1	0.9–1.0	1.5–1.6
Length ratio, caudal setae VII/VI	~ 3	~ 3.5	~ 2.2	~ 3	≥ 1	1.4	~ 2/3
Length ratio, caudal seta V/ramus	~ 1.6	~ 1.4	~ 1	~ 1.3	~ 0.7	~ 0.9	~ 0.9
Leg 3 enp-3 armature formula	4,III	3,III	4,III	3,II	3,III	3,III	3,III
Leg 4, length ratio of inner spine/enp-3	1.5–1.8	~ 1.3	1.6	1.6−1.7	1.2	~ 1.4	~ 1.2
♀ leg 5 exp, length ratio of inner/outer spines	1.2–1.5	~ 1.1	1.2	~ 1.2	0.9	1.1	1.2
♀ leg 5 exp, length ratio of inner/lateral spines	1.3	1.5	1.3	~ 1.3	0.9	1.3	1.4
♀ leg 5, length ratio of inner distal spine/exp	0.9–1	~ 1.2	0.7	0.7–0.8	0.5	0.86	0.86
♂ leg 5, no. of segments	3	3/4 ^†^	-	3	4	3	3
Distribution	Ghana^1^; West Indies^2^	Australia^3^	Cuba^4^	Black Sea^5^	Australia^6^	Brazil^7^	Korea^8^

† incomplete division

References: ^1^ Pleşa (1961), ^2^ Pesce (1985), ^3^ Karanovic (2008), ^4^ Pleşa (1973), ^5^ Pleşa (1964) & Monchenko (1974), ^6^ De Laurentiis et al. (1997) & Karanovic (2008), ^7^ Herbst (1955) & Lotufo and Rocha (1993), ^8^ present study.

### Geographical records and a key to *Neocyclops* species from the Indo-Pacific Ocean

**Pacific Ocean**: from Papua New Guinea, *Neocyclops papuensis* Fiers, 1986 by Fiers (1986).

**Indian Ocean**: from Australia, *Neocyclops australiensis* Karanovic, 2008, *Neocyclops sharkbayensis* Karanovic, 2008, *Neocyclops trajani* Karanovic, 2008 and *Neocyclops tropicus* Karanovic, 2008 by Karanovic (2008); from West Australia, *Neocyclops petkovskii* De Laurentiis, Pesce & Halse, 1997 by De Laurentiis et al. (1997); from Maldive Archipelago, *Neocyclops parvus* (Sewell, 1949) and *Neocyclops magnus* (Sewell, 1949) by Sewell (1949); from Red Sea, *Neocyclops herbsti* Petkovski, 1986, and from Mozambique, *Neocyclops wellsi* Petkovski, 1986 by Petkovski (1986); from Egypt, *Neocyclops salinarum* (Gurney, 1927) by RBINS (2015).

**Table:** 

1	Female antennule 8-segmented	***Neocyclops salinarum***
–	Female antennule 10-segmented	**2**
–	Female antennule 12-segmented	**3**
2	Caudal rami not more than 1.7 times as long as wide in female; male leg 5 3-segmented	***Neocyclops hoonsooi* sp. n.**
–	Caudal rami about 2.4 times as long as wide in female; male leg 5 4-segmented	***Neocyclops petkovskii***
3	Caudal rami less than 1.5 times as long as wide in female	**4**
–	Caudal rami about 2–2.7 times as long as wide in female	**5**
–	Caudal rami 3–3.5 times as long as wide in female	***Neocyclops papuensis***
4	Dorsal caudal seta VII less than twice as long as ramus	***Neocyclops trajani***
–	Dorsal caudal seta VII more than twice as long as ramus	***Neocyclops tropicus***
5	Exopodal seta on antenna lacking	**6**
–	Exopodal seta on antenna present	**8**
6	Inner caudal seta VI longer than outer caudal seta III	***Neocyclops parvus***
–	Inner caudal seta VI shorter than outer caudal seta III	**7**
7	Enp-2 of legs 1–3 with single inner seta; exp-3 of leg 1 with 2 spines	***Neocyclops sharkbayensis***
–	Enp-2 of legs 1–3 with 2 inner setae; exp-3 of leg 1 with 3 spines	***Neocyclops australiensis***
8	Setal formula of exp-3 of legs 1–4 5,5,5,5	***Neocyclops magnus***
–	Setal formula of exp-3 of legs 1–4 5,5,5,4	***Neocyclops herbsti***
–	Setal formula of exp-3 of legs 1–4 4,5,5,5	***Neocyclops wellsi***

## Supplementary Material

XML Treatment for Neocyclops hoonsooi
